# Auditory sensory processing induces cortical and thalamic event-related desynchronization in the mouse

**DOI:** 10.1371/journal.pone.0334293

**Published:** 2025-10-27

**Authors:** Sarah H. McGill, Qilong Xin, Taruna Yadav, Charlie W. Zhao, Patrick Paszkowski, Fabrizio Darby, Mrinmoyee Guha, Tramy Nguyen, David S. Jin, Yuval Nir, Jiayang Liu, Lim-Anna Sieu, Hal Blumenfeld

**Affiliations:** 1 Department of Neurology, Yale University School of Medicine, New Haven, Connecticut, United States of America; 2 Department of Physiology and Pharmacology, Tel Aviv University, Tel Aviv, Israel; 3 Sagol School of Neuroscience, Tel Aviv University, Tel Aviv, Israel; 4 Department of Neuroscience, Yale University School of Medicine, New Haven, Connecticut, United States of America; 5 Department of Neurosurgery, Yale University School of Medicine, New Haven, Connecticut, United States of America; Sorbonne Universite UFR de Biologie, FRANCE

## Abstract

Studies of human perception have shown early cortical signals for primary information encoding, and later signals for higher order processing. An important late signal is the cortical event-related desynchronization (ERD) in the alpha (8–12 Hz) and beta (12–30 Hz) frequency band, which has been linked to human perceptual awareness. Detailed mechanistic investigation of the ERD would be greatly facilitated by availability of a suitable animal model. We conducted local field potential recordings in the mouse frontal association cortex (FrA), thalamic intralaminar centrolateral nucleus (CL), primary auditory cortex (A1), and primary visual cortex (V1) during two auditory tasks. Fully audible brief 50 ms stimuli with both tasks produced early broadband gamma (30–100 Hz) frequency activity at 0–250ms, followed by a late cortical alpha/beta ERD 250–750 ms after stimulus onset. The ERD was statistically significant in FrA and A1, but not in V1. Interestingly, a significant ERD was also observed in thalamic CL. The magnitude of the ERD at full stimulus intensity, and the slope of the relationship between stimulus intensity versus ERD magnitude, were both largest in FrA, and smaller in CL and A1. Conversely, for early broadband gamma activity the magnitude at full intensity and slopes were largest in A1, smaller in CL and smaller still in FrA. These findings strongly support mice as a promising platform for further investigation of the ERD to better understand the origin and function of this robust yet understudied electrophysiological phenomenon.

## Introduction

Conscious perception is a critical feature of normal sensory processing, and its disruption leads to behavioral and cognitive deficits [1–3]. Yet, the neurophysiological origin of sensory processing and conscious perception remain elusive. Early electroencephalographic (EEG) and magnetoencephalographic (MEG) recordings in humans identified decreased cortical alpha (8–12 Hz) and beta (12–30 Hz) activity with both passive sensory stimuli and stimulus-response tasks [4–6]. In the decades following these observations, substantial evidence has related these alpha/beta band event-related desynchronization (ERD) responses with systems underlying perception and higher-order sensory processing. In visual search and discrimination tasks, stimulus-induced ERD amplitudes increase with the number of distractors and with the similarity of target stimuli to distractors [7,8]. In memory tasks, alpha/beta band ERDs are induced with memory retrieval, exhibit differences between hemispheres, and have greater differences in amplitude between hemispheres in semantic versus episodic tasks [9], and in tasks of greater complexity [10]. In studies of volition and motor awareness, transient alpha band ERDs are observed preceding and during un-cued volitional movements [5,11]. Attention-related systems may partly underlie the ERD response [7], a hypothesis supported by several studies which found attention can modulate stimulus- induced ERD [12–15]. Yet, data also support arousal systems in the ERD, including auditory stimulation studies in sleep and anesthesia showing that early primary auditory cortex signals are relatively spared in these states, whereas alpha/beta ERDs are markedly suppressed [16,17].

Despite this important work, data identifying the neural dynamics responsible for alpha/beta ERDs are sparse. The central problem is that studies of alpha/beta ERDs, perception, and of sensory processing more generally [18], are conducted mainly in humans, making testing of mechanistic hypotheses difficult. Our goal was, therefore, to establish an animal model for investigation of sensory processing-related ERDs. Towards this end we sought to assess whether mice exhibit stimulus-induced alpha/beta ERDs, and if so characterize the response. Methods for measuring changes in electrophysiological spectra of neurons are well-established across numerous mammalian species including mice [19]. Yet methods for measuring and manipulating the activity of neuronal subpopulations are more accessible in mice than in other species [20], supported by numerous genetic tools allowing for fundamental investigation of genetically-defined neural populations and specific neurotransmitters. Moreover, despite the anatomical differences between mice and humans, studies have shown early components of sensory processing in both species are highly similar and in many areas nearly identical [21–25].

In this study we recorded local field potentials (LFPs) from the frontal association cortex (FrA), thalamic intralaminar centrolateral nucleus (CL), primary auditory cortex (A1), and primary visual cortex (V1) in mice during passive listening and go/no-go tasks with variable auditory stimulus intensities. A1 and V1 were chosen as targets to enable interpretation of signals uniquely associated with audition. A1 is robustly demonstrated as the cortical center conducting early auditory processing, while V1 is specialized for early visual processing, allowing the former to act as a positive target and the latter a negative control for auditory responses. FrA and CL were chosen as targets to capture signals associated with higher systems of sensory processing across both cortical and subcortical regions. In mice FrA has been linked to cognition [26] and associative learning [27,28]. CL is less well studied in this species, but in other species including humans CL has been linked to acute conscious perception [29–31], arousal [32–35], and attention [33,36]. To our knowledge, no previous studies have identified whether alpha/beta ERDs occur in either of these regions following acute stimulus presentation in an animal model.

Our data confirm that mice exhibit cortical alpha/beta ERDs in FrA and A1 but not V1 between 250 and 750 ms following auditory stimulus presentation. Our results also show the presence of subcortical alpha/beta ERDs within the mouse CL during this same time frame. Of all regions tested, we found that alpha/beta ERD is strongest in the mouse FrA. Moreover, we found that FrA, CL, and A1 exhibited rapid, broadband increases in LFP power between 0 and 250 ms following auditory stimulus presentation, strongest in A1. Finally, we confirm that the mouse visual cortex shows a weak but statistically significant cross-modal early gamma response to auditory stimulus presentation without the alpha/beta ERD. These findings demonstrate the potential for mice as an effective platform for examining the neurophysiological systems underlying this unique electrophysiological phenomenon, and for further investigations of the fundamental nature of the ERD origin.

## Materials and methods

### Animal research ethics

To ensure that animals used in this study received humane treatment, all protocols administered to animals were reviewed and approved by the Yale University Institutional Animal Care and Use Committee (IACUC) before implementation (Protocol Number: 10484). In addition, annual inspections were performed by the Yale University IACUC to independently verify ethical standards were met. To minimize suffering, all animals undergoing surgery were administered isoflurane and ketamine for anesthesia alongside an extended-release buprenorphine formulation for analgesia. Following surgery all animals were further administered Carprofen (brand name Rimadyl) daily for up to 3 days to control pain and inflammation.

### Mice and surgical procedures

All mice employed in this study were C57Bl/6 mice from Charles River. At the time of surgical manipulation, the animals were between 2 and 6 months of age. In total 32 mice were employed in this study, of which 16 were male and 16 were female.

Mice were anaesthetized for surgery using isoflurane inhalation (3% saturation; Covetrus) followed by intraperitoneal injection with a ketamine (90 mg/kg; Covetrus) and xylazine (9 mg/kg; Covetrus) mixture. As ketamine reduces core temperatures in rodents [37], the mice were warmed to 37°C using a heating pad and heat therapy pump (Gaymar T/Pump Localized Therapy System). To provide analgesia both during and after surgical operations, mice were injected subcutaneously with an extended-release buprenorphine solution (3.25 mg/kg; brand name Ethiqa XR; from Ethiqa) before surgery.

To record neural signals from target regions, mice were implanted with 0.15 mm outer diameter insulated stainless steel bipolar twisted-pair local field potential electrodes (E363/3-TW/SPC; Protech International) as well as 1.60 mm shaft diameter ground screws (8IE3639616XE; Protech International). To permit head fixation, the mice were additionally implanted with a custom-fabricated titanium headplate. Stereotaxic coordinates of electrode locations were calculated relative to bregma using The Mouse Brain in Stereotaxic Coordinates Second Edition [38]. For each mouse the electrodes were targeted to up to three of either the frontal association cortex (+2.58 mm AP; + /- 1.39 mm ML; −1.40 mm SI), the thalamic intralaminar centrolateral nucleus (−1.58 mm AP; + /- 0.65 mm ML; −2.72 mm SI), the primary auditory cortex (−2.92 mm AP; + /- 3.83 mm ML; −2.22 mm SI), or the primary visual cortex (−3.40 mm AP; + /- 2.47 mm ML; −1.11 mm SI). A ground screw was targeted to anterior parietal lobe pia, and the headplate was adhered to the skull above the lambdoid sutures with dental cement.

To insert these implants, mice were shaved on their scalps and then mounted onto a stereotaxic frame (Kopf Model 962 Dual Ultra Precise Small Animal Stereotaxic Instrument). Their scalps were then cleaned using alcohol wipes, disinfected with betadine, and injected subcutaneously with lidocaine (0.10 mL at 2% wt/wt; Covetrus) before being excised between the posterior section of the frontal bone plates and anterior section of the interparietal bone plates. The surface of the skull was leveled until the SI positions of bregma, and lambda were within 0.10 mm of each other after which the final locations of both craniometric points were recorded. The target positions of the electrodes and ground screws along AP and ML axes were located using the stereotaxic arm and marked on the skull surface. Burr holes were then made at these locations using a handheld micromotor electrical drill (Rampower). With the brain exposed, the electrodes were inserted to their target depths using a stereotaxic arm as a guide, and the ground screw was driven into its burr hole using a screwdriver to a depth of 1 mm. Dental cement (C&B) was applied to both the electrodes and the ground screws to ensure they were secure.

The headplate was applied to the posterior section of the exposed skull, leveled with a bubble level, and cemented in place. Cement was applied to the edge of the exposed scalp to create a seal between the scalp and skull and prevent infection. Exposed wires from the electrode and ground screws were inserted into threaded cylindrical connectors (Protech International) for interfacing with electrophysiology equipment and covered with acrylic resin for strength and protection (Jet Denture). Postoperatively, mice were injected with carprofen (brand name Rimadyl; 5 mg/ml; from Zoetis) daily for up to three days to prevent inflammation.

### Auditory stimuli and behavioral tasks

Mice were trained in two auditory perception tasks, which both included a brief 50 ms auditory stimulus presented over a range of sound intensities superimposed over a white noise background. Sounds were presented to the mice using speakers (HiVi Tn25 Fabric Dome Tweeter) enclosed in 3D-printed housings flanking the running wheel. Stimuli were generated using a dedicated digital to analog converter and amplifier circuit board (Geekworm X400 V3.0 Audio Expansion Board). The primary auditory stimulus was a complex chord consisting of 17 constant, falling and/or rising sine wave tones distributed between 4KHz and 20KHz frequencies overlaid on each other, with a duration of 50ms. These stimulus features were chosen to ensure the auditory cortex, which in mice is tonotopically organized [39], undergoes spatially widespread activation with above-threshold stimulus presentation. White noise was played continuously as a background mask and to minimize the effects of natural variation in ambient noise. Intensities of the complex chord stimuli and white noise background were measured using a calibrated class 1 sound level meter (DSM403SD; General). The median volume of the ambient noise in the behavioral testing area was 41.6 dB. The median volume of the audio system playing the white noise mask alone was 63.5 dB. The median volume of the complex chord stimulus played at maximum-intensity with the white noise mask was 65.7 dB. For both behavioral tasks the complex chord stimulus was played over a range of fixed intensities modulated by an amplitude scalar, a unitless multiplier applied to the amplitudes of audio file waveform samples to control playback volumes. This scalar ranged from a value of 0 (no stimulus, corresponding to background white noise being played alone at 63.5 dB), to 0.1 (maximum-intensity stimuli, resulting in a total measured volume of 65.7 dB including background white noise).

Behavioral paradigms were administered in automated fashion using a dedicated single board computer (Raspberry Pi 4 4GB) running custom Python software. Using the built-in 5V GPIO terminals, this computer was wired directly to the CED 1401 data acquisition unit to allow the exact timing of all events associated with the behavioral paradigms to be recorded. The two behavioral tasks were a passive listening task where sound stimuli were presented and no response was required, and an auditory go/no-go task where mice were trained to respond to sound stimuli by licking a lick port to receive a sucrose water reward (5% sucrose). Initial analysis of the electrophysiological data from the two tasks showed very similar early broadband activity, and late alpha/beta ERDs for both tasks, so data from the two tasks were combined in the final analyses to maximize sample sizes. Individual mice either underwent the passive listening task alone, the auditory go/no-go task alone, or both tasks in sequence.

Mice were allowed a minimum of 3 days to recover from surgery before acclimatization to the testing apparatus with head fixation while on a running wheel for 30 minutes daily over a minimum of 2 additional consecutive days. The passive listening task required no additional training, but the auditory go/no-go task required an average of 4–5 weeks of training for mice to associate the sound stimulus to the sucrose water reward. Passive listening task sessions ended once 45 minutes had passed, while go/no-go task sessions ended once 45 minutes had passed or the mouse had received its daily allotted quota of water. In both tasks, individual trials consisted of a 2-8s intertrial interval, a 50ms sound stimulus phase, and a fixed 1.5s post-stimulus delay phase. The 2-8s intertrial intervals were characterized by a delay of random length distributed along an exponential curve with a median duration of 4s. The stimulus phases were characterized by presentation of either no auditory stimulus, or presentation of the 50 ms complex chord stimulus at a random volume within the range described above. In the passive listening task the next trial began immediately after the 1.5s post-stimulus delay, whereas in the go/no-go task there was an additional interval of 3.5 s for licking during successful trials and 13.5 s for punishment in unsuccessful trials, before the next trial would commence. Electrophysiological data for both tasks were initially cut into epochs spanning from 1.5s before to 1.5s after each stimulus, and final analyses were performed only on data from 1s before to 1s after each stimulus, so that identical peri-stimulus intervals were analyzed for both tasks.

To evaluate performance in the go/no-go task, we calculated the sensitivity and specificity of the lick response for each session. The sensitivity measured the fraction of lick responses within all maximum-intensity stimulus trials for the session, while the specificity measured the fraction of withhold-lick responses within all no-stimulus trials for the session. To identify the final performance of the mice after training, we calculated the mean sensitivity and specificity across the final five trails administered to the mice.

### Electrophysiology

Local field potential waveforms were amplified by 1000x (A-M Systems Model 1800 Microelectrode AC Amplifier; 0.1 Hz low cutoff, 10kHz high cutoff), before being low pass filtered at 100 Hz using an analog filter (Model 3364 Krohn-Hite). To minimize the effects of mechanical or electrical interference on these recordings, all electrophysiological measurements were made with mice inside a Faraday cage grounded to the building and mounted to an air table. The electrophysiological waveforms were recorded using a CED data acquisition unit (Micro1401−4 with ADC12 expansion unit; CED) at a sampling rate of 1000 Hz.

### Histology

Histology was employed to verify the locations of the electrodes. For this purpose, mice were anaesthetized with isoflurane inhalation and intraperitoneal injection of a ketamine and xylazine mix. They were then euthanized through intraperitoneal injection of 0.05mL of a pentobarbital sodium (at 390 mg/ml) and phenytoin sodium (at 50 mg/ml) solution (brand name Euthasol; from Virbac). To fix the brain tissue, the mice were administered intracardial perfusions using a solution of heparin (1000 units/ml; Sagent Pharmaceuticals) in phosphate buffered saline (PBS; Sigma-Aldrich), followed by a mixture of formaldehyde (4% wt/wt; Thermoscientific) in PBS. The brains were then excised and allowed to sit in a formaldehyde solution for a minimum of 24 hours. After fixation, they were washed in PBS and coronally sectioned into 60µm slices using a vibratome (Leica vt1000s). The slices were mounted onto polarized slides (Epredia) and allowed to dry for 24 hours before being stained with cresyl violet (FC Neurotechnologies). A glass coverslip (Epredia) was secured on top of the stained slices with Cytoseal 60 (Epredia) for protection. The slides were imaged using a Leica DM6 B microscope. All electrophysiological recordings from electrodes implanted in an incorrect location were excluded from analyses.

### Data preprocessing

All data were analyzed using MATLAB (R2023b; Mathworks). The MATLAB/SON interface library provided by CED was used to open and read from the electrophysiological recording files in MATLAB. Individual trials with LFP recording amplitudes over 500µV in at least one sample were rejected as outliers. For any condition, only animals with at least ten trials applicable to the condition were included in analyses to maximize the signal-to-noise ratio in the results. Exact timings of auditory stimuli were determined by analyzing the audio channel waveforms generated by the digital to analog converter and amplifier board and recorded by the CED data acquisition unit. For this purpose, waveform elements exceeding 3.5 standard deviations from the median amplitude and occurring within 10ms of each other were clustered. Clusters shorter than 0.03s or longer than 0.07ms, or those occurring outside the 1.55s span following a TTL pulse marking execution of the stimulus presentation function, were considered erroneous and excluded. The stimulus start time was defined as the timestamp of the first sample in each valid cluster. In trials where no stimulus was presented or when low-intensity stimuli were indistinguishable from background noise, a fallback estimate of 119.3ms after stimulus function execution was used. This estimate was derived from diagnostic tests measuring the median delay between the TTL pulse and stimulus onset for maximum-intensity complex chord stimuli. After preprocessing, recordings from each trial were cut into 3-second epochs spanning the 1.5 seconds before and after the auditory stimulus onset.

### Time-frequency analysis

Time-frequency spectrograms were computed from LFP recordings aligned to stimulus onset. Using a fast-Fourier implementation (spectrogram function in MATLAB) of a discrete Fourier transformation (DFT), power was computed in 250ms time windows with 218ms overlap. The spectrogram power values at each time point and frequency were converted to decibel scale ($\text{1}\text{0}\times {\text{log}}_{\text{1}\text{0}}\frac{(Power}{Baseline)}$), where baseline was defined for each frequency as the mean power over the interval from 500ms prior to auditory stimulus onset until the point just prior to stimulus onset. These time-frequency spectrogram values were then pooled within each animal by averaging across trials at each stimulus intensity for downstream analysis.

**Fig 1:**
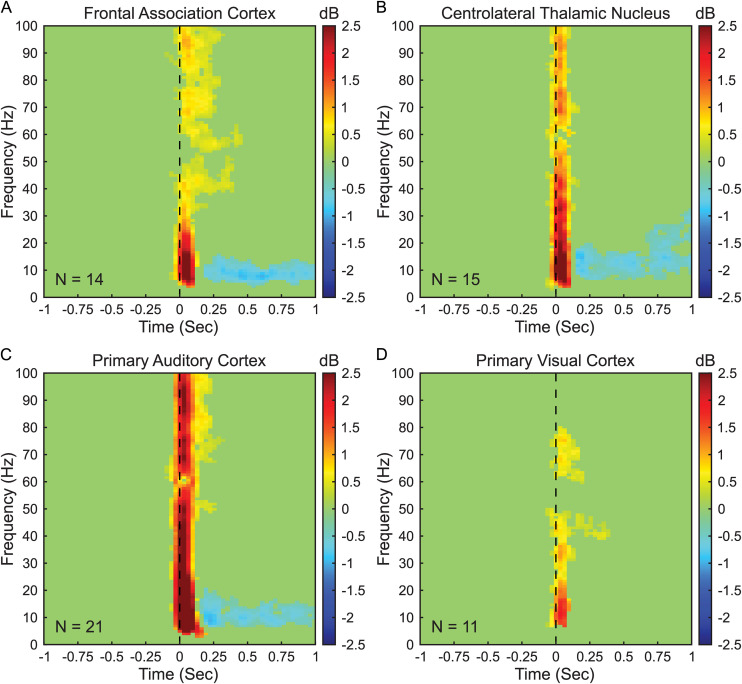
Cortical and subcortical regions feature distinct early and late responses during audition. The differences between the mean peristimulus spectrograms of maximum-intensity auditory stimulus trials (65.7 dB) and no-stimulus trials (63.5 dB) are shown here. Spectrograms time-locked to real or no-stimulus (blank) onset (time 0, vertical dashed line) were computed for all maximum-intensity and no-stimulus trials, then decibel-normalized to the prestimulus baseline (500ms before stimulus presentation). Peristimulus spectrograms were averaged across all trials of the same condition for each mouse, then the mean spectrogram across all mice was found for all trials of the same condition. The mean no-stimulus trial spectrograms were subtracted from the mean maximum-intensity trial spectrograms, and a cluster-based permutation testing was employed to identify significant differences between the conditions (p < 0.05, 5000 iterations). Warmer tones indicate increases in spectral power within maximum-intensity stimulus trials over no-stimulus trials, while cooler tones indicate decreases. **(A)** Frontal association cortex, (B) centrolateral thalamic nucleus, and (C) primary auditory cortex show early increases in alpha (8-12 Hz), beta (12-30 Hz), and gamma (30-100 Hz) band frequencies with late decreases in alpha and beta frequency bands. **(D)** Primary visual cortex shows only increases to early alpha, beta, and gamma band power. Number of mice (N) are shown for each panel.

To identify time-frequency changes associated with presentation of auditory stimuli, we compared maximum-intensity trials (65.7 dB total, with 50 ms maximum-intensity complex chord stimulus plus white noise background mask) to no-stimulus trials (63.5 dB, with white noise background mask only). Statistical significance of the differences between these two conditions was determined using a cluster-based permutation test (see next section). The time-frequency response associated with stimulus presentation in each recorded region was identified by calculating the difference between the maximum-intensity and no-stimulus conditions taken as the mean across mice (see Fig 1).

### Cluster-based permutation testing

Maximum stimulus-intensity peristimulus spectrograms were compared to no stimulus trial spectrograms using hypothesis-free cluster-based permutation testing. This approach has been described previously [30,40]. Briefly, a null distribution was created by randomly shuffling data between the maximum stimulus intensity and the no stimulus conditions across mice (n = number of animals). A first level two-tailed t-test was performed comparing maximum stimulus intensity and no stimulus conditions, and the associated t-values were calculated for each time point and frequency. These t-values were used to cluster the data based on frequency and temporal adjacency, defined as the neighboring frequency and time points respectively. Only those clusters whose sum of t-values were highest were kept for each iteration. This shuffle and clustering process was repeated with 5000 iterations to create a null distribution. Finally, a two-tailed t-test was performed on the actual data of the maximum-intensity and no-stimulus intensity conditions and similarly clustered based on frequency-time adjacency. Only those clusters with sum of t-values higher than the 95th percentile of the null distribution were considered statistically significant. This approach was designed to address the multiple comparison problem associated with comparing data across large numbers of time points and frequencies. A modified version of the clust-perm1 function from the MATLAB Mass Univariate ERP Toolbox created by David Groppe [40] was employed to conduct this analyses. Finally, to remove small spurious clusters, only those clusters exceeding 89 time-frequency elements (1% of the total number of time-frequency elements in the spectrogram) in size were kept.

### Power spectral density analysis

**Fig 2:**
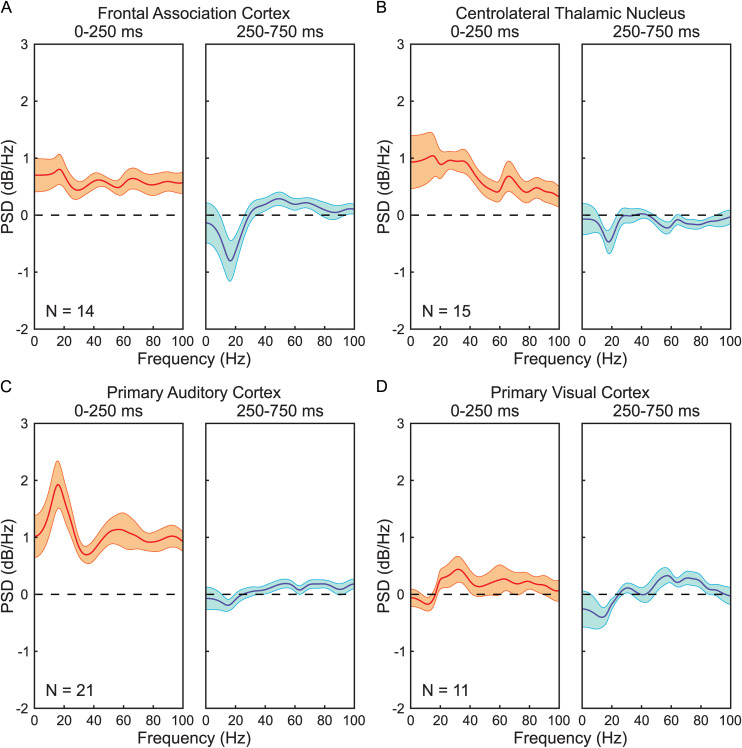
Spectral compositions of local field potentials shift during audition from broadband increases to alpha-beta band desynchronizations. The differences between mean power spectral density (PSD) curves for maximum-intensity auditory stimulus trials (65.7dB) and no-stimulus trials (63.5dB) within early (0-250ms) and late (250-750) postimulus periods are shown here. PSD curves were computed for early and late postimulus windows in all maximum-intensity and no-stimulus trials. PSDs were then decibel normalized to their respective prestimulus baselines. For each mouse and recording site PSDs were averaged across all mice within each condition, and the mean PSDs for no-stimulus condition were subtracted from the mean PSDs for the maximum-stimulus condition. The differences in PSDs were then averaged across all mice. Positive values indicate greater spectral density at corresponding frequencies for maximum-intensity trials than no-stimulus trials, while negative values indicate the opposite. Presentation of auditory stimuli results in broadband increases to (A) mouse frontal association cortex, (B) centrolateral thalamic nucleus, and (C) primary auditory cortex with peaks in alpha (8-12 Hz) and beta (12-30 Hz) frequency bands during the early post-stimulus period. In the late post-stimulus period, prominent alpha-beta desynchronizations are found in the frontal association cortex and centrolateral thalamic nucleus, with smaller changes in primary auditory cortex. **(D)** Primary visual cortex shows limited increases to LFP power in mainly beta and low gamma (30-100 Hz) bands during the early post-stimulus period. Center lines plot mean of PSDs across mice. Upper and lower bands show standard error of the mean. Number of mice (N) are shown for each panel; same animals and trials as in Fig 1. Note that for Fig 2 all changes are shown without thresholding; for statistically significant changes please see Fig 1.

Power spectral densities (PSDs) of peristimulus recordings were obtained for each trial using Welch’s method applied to two time-windows of interest: the early post-stimulus period (0-250ms following stimulus onset) and the late post-stimulus period (250-750ms post-onset). This same method was also used to calculate the PSDs of the prestimulus baselines (defined as the 500ms prior to stimulus onset). These computations were performed using a 100 ms Hamming window with 50% overlap between segments. The PSDs were next aggregated within each animal by calculating the mean PSD for each target brain location, time window, and sound intensity across all relevant trials. The PSDs of each type for each animal were then decibel normalized ($\text{1}\text{0}\times {\text{log}}_{\text{1}\text{0}}(\frac{Power}{Baseline)}$) to their corresponding mean baseline PSDs, with the baseline interval as already defined. The no-stimulus condition PSDs were then subtracted from their corresponding maximum-intensity stimulus condition PSDs for each combination of brain location and time window. Finally, the PSDs were combined across animals by identifying the average PSD difference between maximum-intensity and no-stimulus conditions within each brain location and time window (see Fig 2).

### Stimulus intensity regression

**Fig 3:**
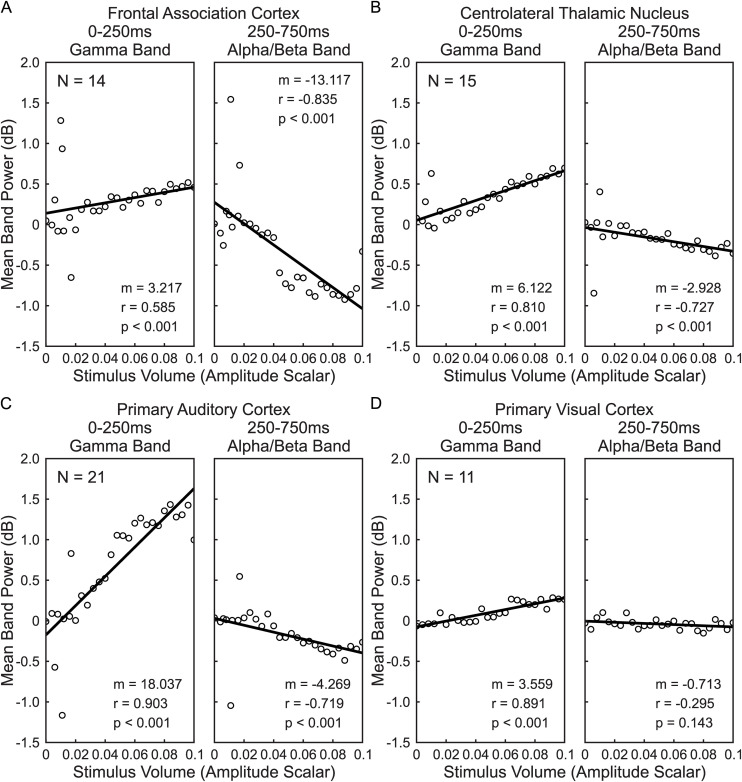
Stimulus intensity predicts early and late auditory response amplitudes. The relationship between early gamma band power (0-250ms, 30-100 Hz) and late alpha/beta band power (250-750ms, 8-30 Hz) are shown for each recorded brain region. Spectrograms centered around stimulus onset were computed for each trial and decibel-normalized to the 500ms prestimulus baseline. The mean early gamma band power (0-250ms, 30-100 Hz) and late alpha/beta band power (250-750ms, 8-30 Hz) were then found for each trial. Within each mouse the average early gamma and late alpha/beta band powers were calculated for all stimulus intensities, and a linear model was fit to the resulting distribution. The mean band power across all mice was calculated for each stimulus intensity and is plotted here. Stimulus intensity values shown are scalar multipliers of the 50ms complex chord audio waveform samples, and in units such that 0 corresponds to no sound, and 0.1 corresponds to the maximum sound stimulus used. Mouse frontal (A) association cortex, (B) centrolateral thalamic nucleus, (C) primary auditory cortex, and (D) primary visual cortex exhibit significant positive relationships between early post-stimulus gamma power and stimulus intensity as well as between late post-stimulus alpha/beta power and stimulus intensity, although the strength of these relationships varies. Spearman correlation coefficient **(r)**. Significance of correlation **(p)**. Regression slope **(m)**. Number of mice (N) are shown for each panel; same animals as Figs 1 and 2 but using variable stimulus intensity trials.

To identify the effect of stimulus intensity on the spectral composition of electrophysiological responses elicited from stimulus presentation, a regression analysis of selected time-frequency bands against stimulus intensity was performed. The selected time-frequency bands consisted of the gamma band (30–100 Hz) of the early post-stimulus period (0–250ms) and the alpha/beta band (8–30 Hz collectively) of the late post-stimulus period (250–750ms). Towards this end, the subset of the peristimulus time-frequency spectrograms within the selected time-frequency bands were extracted for each trial following the baseline-corrected decibel normalization as detailed above. We then calculated the mean values across all frequency and time points within the selected time-frequency bands for each trial. Following this, the mean early gamma band and the mean late alpha/beta band responses were calculated within each animal for each target brain region across all trials of the same intensity. Linear models were fit to stimulus intensity versus either early gamma band power or late alpha/beta band power for each target brain region, treating mice as replicates (see Fig 3). The strength of this relationship was identified by calculating Spearman’s rank correlation coefficient, and the slope of the linear fit. The significance of fit was identified by calculating the p-value associated with the coefficient of linear regression.

## Results

To characterize mouse brain activity across cortical and subcortical regions during auditory perception, we measured local field potentials (LFPs) within the frontal association cortex (FrA), thalamic centrolateral nucleus (CL), primary auditory cortex (A1), and primary visual cortex (V1) of mice during passive listening and auditory go/no-go tasks featuring stimuli of variable intensity. We then evaluated the behavioral outcomes of auditory go/no-go sessions during trials with maximum-intensity (65.7 dB with both stimulus and white noise background) stimuli as well as trials with no-stimulus (63.5 dB with white noise background only) to identify if licking behavior reliably predicted acute conscious perception. The mean sensitivity, or the probability that a mouse will lick for a maximum-intensity stimulus, calculated for the last five sessions for each mouse and averaged across all animals was 0.886 ± 0.041 SEM. The mean specificity, or the probability a mouse will not lick given no stimulus was presented, calculated using an identical approach was 0.390 ± 0.037 SEM. Because licking behavior did not reliably distinguish perception from non-perception, we next asked whether electrophysiological responses in passive listening and go/no-go tasks were distinct. We compared the baseline-normalized spectrograms from maximum-intensity stimulus trials to the no-stimulus trials in each task using a cluster-based permutation test, and found few qualitative differences between the spectral distributions of go/no-go task trials and passive listening task trials, particularly for the alpha/beta ERD (S1 Fig). As a result, we chose to pool data from each task in subsequent analyses to increase sample size.

To identify time-frequency power changes associated with stimulus presentation within the recorded regions, we sought to identify signals present in trials with maximum-intensity stimuli but absent from trials with no stimuli at comparable time frames. For each maximum-intensity stimulus trial we found the peristimulus spectrogram centered around stimulus onset, and for each no-stimulus trial we found the spectrogram centered around the no stimulus (blank) onset time. We decibel normalized each spectrogram to the spectral distribution of a 500ms prestimulus baseline from the same trial. Then, for each mouse we calculated the mean spectrogram across all trials within the same stimulus condition. We computed the difference between the mean maximum-intensity and no-stimulus trial spectrograms for each mouse, and identified the mean difference across all mice. Finally, we employed cluster-based permutation to highlight time-frequency clusters which were significantly different between maximum-intensity stimulus and no-stimulus trials in each task. This approach revealed that presentation of auditory stimuli results in significant (p < 0.05) changes to LFP power in all brain regions targeted (Fig 1).

FrA, CL, and A1 showed temporally bimodal responses with distinct early (0−250ms) and late (250−750ms) response components. The early response components were characterized by increases in LFP power spanning alpha (8−12 Hz), beta (12−30 Hz), and gamma (30−100 Hz) frequency bands. Following stimulus onset and up to 250ms post-stimulus, the FrA (Fig 1A) exhibited a + 0.98 dB mean increase in alpha band power, a + 0.69 dB increase in beta band power, and a + 0.44 increase in gamma band power. The CL (Fig 1B) in this same time frame showed a + 0.78 dB increase in alpha band power, a + 0.76 dB increase in beta band power, and a + 0.53 dB increase in gamma band power. And the A1 (Fig 1C) in the early response period featured a + 1.84 dB increase in alpha band power, a + 1.49 dB increase in beta band power, and a + 1.08 dB increase in gamma band power. The late response components within these regions consisted of a decrease to both alpha and beta band power that is characteristic of alpha/beta event-related desynchronization (ERD). The FrA (Fig 1A) during this period showed a −0.80 dB decrease to alpha band power and a −0.24 dB decrease to beta band power. The CL (Fig 1B) in this same interval exhibited a −0.52 dB decrease to alpha band power and a −0.35 dB decrease to beta band power. And the A1 (Fig 1C) during the late response period featured a −0.59 dB decrease to alpha band power and a −0.24 dB decrease to beta band power.

Interestingly, the V1 (Fig 1D) also exhibited a response to presentation of maximum-intensity auditory stimuli significantly different from no-stimulus controls. Like the FrA, the CL, and the A1, the V1 featured a broadband increase in LFP band power within 250ms of stimulus onset. Yet, the increase in LFP band power observed in the V1 was weaker than those found in other regions. During the early post-stimulus response period V1 showed a + 0.06 dB increase in alpha band LFP power, a + 0.18 dB increase in beta band LFP power, and a + 0.28 dB increase in gamma band power. Moreover, no significant difference in LFP band power was identified between maximum-intensity stimulus trials and control no-stimulus trials following this initial response.

While spectrographic analyses expose the exact times that changes in the spectral composition of LFP signals occur, sacrifices in frequency resolution imposed by the technique prevent the exact frequency content of any underlying signals from being precisely identified. To accurately characterize the spectral content of the early (0–250ms) and late (250–750ms) postimulus response components, we computed the average power spectral density (PSD) of the peristimulus LFP recordings over these respective time windows. Towards this end we first calculated PSD curves for maximum-intensity and no-stimulus trials within the early and late postimulus time windows. We then decibel normalized the PSDs of each trial relative to the PSDs of their prestimulus baselines. For each mouse and time window we computed the mean PSD within each stimulus condition, and then we identified the difference between conditions by subtracting the mean no-stimulus PSD from the mean maximum-intensity PSD. Finally, we identified the average difference in PSD spectra between trial conditions across all mice.

In the early post-stimulus period the FrA (Fig 2A, left), CL (Fig 2B, left), and A1 (Fig 2C, left) showed broadband increases to LFP PSD compared to intertrial interval baselines. The mean change in LFP PSD across all frequencies in the FrA, CL, and A1 were found to be + 0.60 dB/Hz, + 0.86 dB/Hz, and +1.22 dB/Hz respectively. In addition to the broadband changes, these regions showed peaks in the alpha and beta frequency ranges. The greatest magnitude of the early LFP post-stimulus response in the FrA was found to be at 17 Hz with a PSD peak of +0.80 dB/Hz, the maximum in the CL was identified at 15 Hz with a peak of +1.04 dB/Hz, and the maximum in the A1 was determined to be 16 Hz with a peak of +1.93 dB/Hz. In V1 (Fig 2D, left), there were also increases in LFP PSD during the early post-stimulus response period, although these increases were limited mainly to the beta and low gamma frequency bands, with a peak of +0.44 dB/Hz at 32 Hz.

In the late post-stimulus period, FrA (Fig 2A, right) and CL (Fig 2B, right) showed substantial decreases to alpha and beta band LFP PSD. Within this time span, the response was not broadband, but instead localized to lower frequencies. Prominent minima were found during the late post-stimulus period in the FrA at 17 Hz with a decrease of −0.81 dB/Hz and in the CL at 18 Hz with a decrease of −0.47 dB/Hz, and a smaller minimum was found for A1 (Fig 2C, right) at 15 Hz with a decrease of −0.19 dB. In V1 (Fig 2D, right), we observed a minimum at 14 Hz with a decrease of −0.40 dB/Hz in the late post-stimulus period, although this change did not reach statistical significance with time-frequency permutation-based clustering analysis.

To characterize how response magnitude and spectral composition changed with sound level, we varied the intensity of target stimuli presented to mice within both passive listening and go/no-go tasks. For each trial we calculated the peristimulus spectrogram centered around stimulus onset and decibel normalized the spectrograms relative to their 500ms prestimulus baselines. We then computed the mean early gamma band power (0–250ms & 30–100 Hz) as well as the mean late alpha/beta band power (250–750ms & 8–30 Hz) for each trial. For each mouse we found the average early gamma band power and late alpha/beta band power across all trials of each stimulus intensity, and then found the average across all mice. Finally, we fit linear models relating stimulus intensity to band power for each of the recording regions.

Regression of LFP power against stimulus intensity revealed significant relationships between these variables in each brain region, time and frequency range of interest, although the slope of these relationships was stronger for some than others (Fig 3). For early post-stimulus period gamma band power, A1 (Fig 3C, left) exhibited the strongest response to changes in stimulus intensity with a slope of 18.037 dB/Scalar Amplitude (p < 0.001) and a Spearman correlation coefficient of 0.903. Thalamic CL (Fig 3B, left) showed the next strongest response within this time and frequency span having a slope of 6.122 dB/Scalar Amplitude (p < 0.001) and a Spearman correlation coefficient of 0.810. The FrA (Fig 3A, left) and V1 (Fig 3D, left) both exhibited smaller slopes in early post-stimulus period, with the FrA exhibiting a slope of 3.217 dB/Scalar Amplitude (p < 0.001) and Spearman correlation coefficient of 0.585, while the V1 exhibited a slope of 3.559 dB/Scalar Amplitude (p < 0.001) and a Spearman correlation coefficient of 0.891.

For the late post-stimulus period alpha/beta band power, FrA (Fig 3A, right) showed the greatest response to stimulus intensity changes with a slope of −13.117 dB/Scalar Amplitude (p < 0.001) and a Spearman correlation coefficient of −0.835. The CL (Fig 3B, right) and A1 (Fig 3C, right) exhibited weaker but still significant responses, with the CL having a response slope of −2.928 dB/Scalar Amplitude (p < 0.001) and a Spearman correlation coefficient of −0.727 and the A1 having a response slope of −4.269 dB/Scalar Amplitude (p < 0.001) and a Spearman correlation coefficient of −0.719. No significant relationship between stimulus volume and late post-stimulus alpha/beta band power was found in V1 (Fig 3D, right; p = 0.143).

## Discussion

To assess the potential for mice as a model of the alpha/beta ERD, we recorded LFP signals in mouse frontal association cortex (FrA), centrolateral thalamic nucleus (CL), as well as in primary auditory and visual cortex (A1 and V1) during passive listening and auditory go/no-go tasks with stimuli of varying intensities. This approach allowed for the isolation of electrophysiological signals created following presentation of auditory stimuli with excellent spatial specificity, high temporal resolution, and within the same frequency ranges employed in humans for studying alpha/beta ERDs. Our results demonstrate that mice exhibit cortical auditory-induced decreases in alpha (8–12 Hz) and beta (12–30 Hz) band LFP activity at 250–750 ms after stimulus onset, analogous to alpha/beta ERDs previously demonstrated in humans. We also report the novel observation that the mouse CL, a component of the intralaminar thalamus [41], exhibits auditory-induced alpha/beta ERDs in a time frame comparable to those found in the mouse cortex. The ERD magnitude and relationship to auditory stimulus intensity was strongest in the association cortex area FrA, and was smaller but still notable in primary auditory cortex A1 and in thalamic CL. Finally, we found that the auditory-induced alpha/beta ERDs identified in the mouse were preceded by widespread, transient increases in broadband LFP signals, consistent with prior work showing broadband cortical signals related to local population neuronal activity [42–46]. In contrast to the ERD, this early broadband signal was largest and most strongly related to auditory stimulus amplitude in A1, intermediate in CL and FrA, and smallest in primary visual cortex V1. Taken together these findings highlight the efficacy of mice as a model organism for studying alpha/beta ERDs, demonstrating that application of existing methodologies can both replicate and extend prior observations found in humans within this more technically versatile species.

### Cortical event-related desynchronization

Time-frequency analysis of peristimulus LFP recordings within maximum auditory intensity trials of passive listening and go/no-go tasks revealed that mouse FrA and A1, but not V1, exhibited statistically significant decreases in alpha and beta band LFP oscillations between 250ms and 750ms post-stimulus. These results confirm that mice exhibit auditory-induced cortical desynchronizations analogous to those previously identified in humans. Auditory-induced cortical ERDs were among the first observations in humans describing the existence of the ERD electrophysiological response [4]. This original study reported decreases in cortical alpha band activity following presentation of auditory stimuli, similar to the decreases found here in mice, and further demonstrated that the ERD could be seen in the context of both passive listening and stimulus-response tasks, a result that was also replicated here through examination of passive listening and go/no-go tasks with mice. Later work expanded on these observations, establishing that auditory-induced cortical ERDs in humans could extend into the beta band [47–49], an observation which aligns with those in our study, and that these responses were measurable using intracranial field potential recordings [31,50–52] similar to those used here. Interestingly, human intracranial recordings showed that alpha/beta ERDs induced by passive auditory stimulation are significantly smaller in sleep and under anesthesia, whereas early activity in primary auditory cortex is relatively spared in these states, leading to the proposal that the alpha/beta ERD represents higher order feedback necessary for conscious perception of sensory stimuli [16,17].

Uniquely, we found that the amplitude of the mouse alpha/beta ERDs was greater in the FrA than in any other region examined, and we discovered through regression of the mean LFP within the alpha/beta range against stimulus intensity that the alpha/beta ERD identified in the mouse FrA was substantially more sensitive to changes in stimulus intensity than any other region. This was somewhat unexpected as in humans auditory-induced alpha/beta ERDs are typically highest in amplitude over central or parietal regions, with amplitudes over frontal and temporal regions being comparable [53–55]. The reason for this discrepancy may lie in differences between mouse and human cortical cytoarchitecture. Electrical deflections measured by EEG, the most common method for measuring extracellular potentials including ERDs in humans [6,56,57], as well as by LFP, the most common method in mice [58,59], are predominantly driven by excitatory postsynaptic potentials in pyramidal neurons [60,61]. Comparative analysis of human and mouse neuroanatomy has revealed notable differences in pyramidal neuron anatomy between these two species. Human pyramidal cells tend to have large somas [62], more and larger dendritic branches [63,64], and a greater number of synapses than those in mice [65]. Moreover, pyramidal neurons in these species have distinct input resistances, time constants, and length constants [66,67]. As the distribution of electrical potentials and overall dipole strength are highly dependent on all of these features [61], it is likely that they contribute to the observed differences in ERD amplitude and spatial distribution between mice and humans.

These divergences underscore a long-standing question within comparative neuroanatomy and rodent neuroscience research. To what extent does the mouse cortex accurately model the human cortex? Literature suggests that there is strong functional analogy between these species in visual cortex [23,68], auditory cortex [69,70], somatosensory cortex [71,72], and both primary and secondary motor areas [73–75]. Yet, the prefrontal cortex presents a problem. While mice exhibit regions which fit the original and still-prevalent definition of prefrontal cortex based on connectivity with the mediodorsal nucleus of the thalamus [76,77], these regions lack the prominent granular layer present in the human prefrontal cortex [78,79] and exhibit distinct thalamocortical connectivity [80]. These differences affect the functions of the prefrontal cortex. The mouse prefrontal cortex may mediate attention [81,82], behavioral inhibition [83,84], and social cognition [85,86] much like the human prefrontal cortex, but it does not display the same responsibility for language [87,88] or analogical reasoning [89] the region exhibits in humans. Differences can also be observed with oscillatory signals. In humans posterior alpha band oscillations increase with eye closure and are blocked with eye opening [90,91]. Comparable experiments performed in mice fail to demonstrate this same phenomenon [92,93]. Collectively these observations suggest that although alpha/beta event-related desynchronization can be seen in both mice and humans using similar behavioral tasks, we cannot conclude from this result alone that the underlying systems mediating the response are comparable.

### Subcortical event-related desynchronization

Given the central role that the thalamus has in coordinating cortical rhythms through cortical-subcortical loops, it is natural to question whether the subcortical regions as well as cortical ones exhibit auditory-evoked alpha/beta ERDs. In this study, we confirm that the CL nucleus of the intralaminar thalamus exhibits alpha/beta ERDs within the same time frame as cortical regions. This result highlights the benefit of employing intracranial LFP measures of extracellular electrical potentials within mice for investigating the neurophysiological systems responsible for generating alpha/beta ERDs and their respective functions. In humans literature studying ERDs almost exclusively rely on surface EEG or MEG as their primary method for examining electrophysiological activity in the brain [6]. These approaches are effective at capturing cortical sources of activity [94], but they are less useful for identifying subcortical sources [95,96]. Alternative approaches which are more effective for assessing electrical potentials in subcortical areas, such as intracranial EEG, exist and have been employed in the study of ERDs in this species [16]. However technical and ethical considerations limit widespread usage of these techniques, and to our knowledge few studies have used them to examine ERDs in the thalamus.

What is the potential function of auditory-induced alpha/beta ERDs within the intralaminar thalamus? We found that the mean alpha/beta LFP power in the CL was negatively correlated with stimulus intensity, or that ERD amplitude increased with stimulus volume. This suggests that the systems driving alpha/beta ERDs in the intralaminar thalamus are engaged with sensory processing. Existing literature supports this conclusion, and further suggests that the intralaminar thalamus and CL are linked to higher-order features of sensory processing [29,32,33,36]. Early studies seeking to identify functions for thalamic regions show that disruption of activity in the intralaminar thalamus through lesion [35] or electrical stimulation [34] results in loss of arousal and conscious awareness. More recent work establishes that neurons in this region exhibit evoked potentials in response to presentation of visual [97,98] or auditory stimuli [97,99]. And critically, presentation of perceived stimuli but not of identical unperceived stimuli results in increases to intralaminar thalamic neural activity [30,31]. Given that intralaminar thalamic nuclei including CL exhibit extensive connections with the cortex [100] mediating synchronization and desynchronization of cortical regions [101–103], these findings suggest that alpha/beta ERD within the CL may represent modulation of cortical systems underlying higher-order sensory processing. Because the present studies were limited to CL as only one thalamic nucleus of interest, to support broader conclusions about thalamic involvement in sensory and perceptual processing, it will be important in future studies to include additional thalamic nuclei, such as the mediodorsal (frontal/limbic), ventral anterior/ventral lateral (motor) or lateral/medial geniculate (visual/auditory) nuclei as comparative controls.

### Transient broadband LFP activity increases

Alongside alpha/beta ERDs, we also found that auditory stimulation resulted in broadband increases to LFP within the mouse FrA, CL, and A1 spanning the 250ms following stimulus presentation. Regression analyses revealed that the mean gamma power in each of these regions was positively correlated with stimulus intensity, and that the A1 showed the greatest sensitivity to changes in stimulus intensity of all regions measured. These observations agree with current understanding of audition and auditory cognition. The necessity of the A1 for audition and the sensitivity of field potentials within the region to auditory stimulation is well established in rodents [104–106]. In FrA single-unit recordings show that neurons respond to auditory stimulation [107], and lesion and conditioning studies demonstrate FrA’s function for auditory learning [27,28,108,109]. Similarly, single-unit activity recordings show that neurons in the CL respond to auditory stimulation [97], and stimulation studies further demonstrate that activity in the intralaminar thalamus mediates cortical synchrony [101,102] including within auditory regions [103]. Broadband enhancement of LFPs has been linked to concurrent increases in neuronal firing rates across local neuronal populations [42–46,110,111]. Our observation of such signals within cortical and subcortical regions that are well-known to contribute to different aspects of audition, reinforces the validity of our novel findings.

Interestingly, we also found that V1 exhibits a broadband increase to LFP within the 250ms span following stimulus presentation. As in other regions we measured, regression analyses within the V1 revealed the mean gamma band LFP power of this early response was positively correlated with stimulus intensity. While a superficial examination of this result might lead to the conclusion that it is erroneous or unexpected, as V1 is traditionally associated with visual and not auditory processing, prevailing theories of visual cortex function hold that the visual cortex integrates information from multiple sensory modalities [112–114]. Indeed, the cross-modal sensitivity in visual cortex neurons to auditory stimuli was noted in early studies of this region [115–117], and more recent work has reported auditory-induced field potential changes comparable to those found here [118,119]. Anatomical studies further show that the mouse V1 receives projections from the auditory cortex [120,121], potentials within which are sufficient for generating LFP deflections [122–124]. Synthesized together, these findings suggest the responses to auditory stimuli found in the visual cortex in this study is not artifactual but reflect another example in the growing literature of cortical cross-modal processing.

### Limitations and future directions

To explore the functional origins of alpha/beta ERDs within mice, we sought to implement an auditory go/no-go task with variable stimulus intensities. This experimental design mirrors auditory discrimination tasks performed in humans [30,31,51], which are used to identify electrophysiological signals associated with higher-order sensory processing and acute conscious perception. Although we successfully trained mice to lick the reward spout following stimulus presentation, the mice failed to reliably withhold licking in its absence, likely due to the consistent delayed presentation of a motorized water spout for all trials in our paradigm. As a result, the mice exhibited insufficient specificity for performing analyses comparable to those in human studies. Possible explanations for this issue include failure to implement a sufficiently aversive punishment to discourage mice from incorrect licking behavior, making the reward too appetitive through the addition of sugar, or inadvertently training mice to respond to the sound of the motorized lickspout presentation rather than the preceding target stimulus. Future studies may seek to resolve this issue, and thereby assess the role of alpha/beta ERDs in auditory perception, however the current study has focused on more basic aspects of auditory processing without drawing direct conclusions about conscious perception in the present data.

An equally important challenge for the interpretation of findings in this study are the fundamental differences in functional neuroanatomy between mice and humans. Most experiments examining alpha/beta ERDs have performed in human subjects [6], which as discussed previously exhibit distinctions from mice in cortical cytoarchitecture [62–65], frontocortical anatomy [76–80] and functionality [87–89], as well as systems controlling alpha band oscillations [90–93]. Speculation on how these features might affect alpha/beta ERDs in mice is possible, but addressing this point with certainty will require additional research.

It is also critical to recognize the limitations in employing LFP recording as a measure of neural activity. Unlike scalp EEG, the standard technique for measuring extracellular potential oscillations including ERDs in humans [6,56,57], LFP recordings are performed using electrodes implanted within neural tissue thus capturing signals at higher spatial resolution [125,126]. This makes LFP preferable to EEG for evaluating extracellular potentials in mice. Yet, attributing LFP signals to their respective sources is notoriously difficult. Experimental validation of LFP recording methodologies confirm that while the predominant source of electrical potentials measured by LFP electrodes originates close (<250µm) to the tip [127,128], in some cases sources centimeters away can contribute [126]. Along these same lines terminal axonal projections have been found to generate electrical fields measurable by LFP methodologies [122–124], and such structures do not necessarily originate from neuronal populations within the region targeted. Further, single neurons exhibit complex patterns of electrical polarization [124] often leading to more complex field distributions when in close proximity [61]. To overcome ambiguities with LFP, future studies may employ additional methods of measuring neural activity including multiunit activity (MUA) or single unit activity (SUA) measures in conjunction with LFP.

With these limitations in mind, it is still remarkable that we found mice exhibit auditory-evoked decreases to cortical alpha and beta band field potentials similar to those previously reported in humans. While additional studies are required to make definitive conclusions, the presence of this phenomenon in mice hints that there may be common systems underlying this response between the species. We also observed auditory-evoked alpha/beta event-related desynchronization within the mouse centrolateral thalamic nucleus, confirming that this phenomenon is not limited to cortical regions. This result highlights the methodological advantages of employing mice as a model organism in this context. Finally, we report widespread, transient broadband increases to local field potential oscillations within cortical and subcortical regions of the mouse brain. These data conform with modern theories of audition and auditory cognition, validating our more novel findings. Considered together, despite the interpretive limitations of local field potential measures of neural activity, this study supports the use of mice as a promising model for investigating the role and systems underlying event-related desynchronizations. Future studies undertaking these endeavors may find the numerous techniques available in this model organism, such as multi-unit and single-unit activity recordings or fiber photometry and optogenetics, as well as behavioral studies critical for understanding the function and purpose of event-related desynchronizations within the brain.

## Supporting information

S1 Fig Passive listening and go/No-go tasks feature comparable electrophysiological responses. The differences between the mean peristimulus spectrograms of maximum-intensity auditory stimulus trials (65.7 dB) and no-stimulus trials (63.5 dB white noise only) are shown here for passive listening (left) and go/no-go (right tasks). Spectrograms time-locked to real or no-stimulus (blank) onset (time 0, vertical dashed line) were computed for all maximum-intensity and no-stimulus trials, then decibel-normalized to the prestimulus baseline (500ms before stimulus presentation). Peristimulus spectrograms were averaged across all trials of the same condition for each mouse, then the mean spectrogram across all mice was found for all trials of the same condition. The mean no-stimulus trial spectrograms were subtracted from the mean maximum-intensity trial spectrograms, and a cluster-based permutation testing was employed to identify significant differences between the conditions (p < 0.05, 5000 iterations). Warmer tones indicate increases in spectral power within maximum-intensity stimulus trials over no-stimulus trials, while cooler tones indicate decreases. (A) Frontal association cortex, (B) centrolateral thalamic nucleus, (C) primary auditory cortex and (D) primary visual cortex show few qualitative differences in between passive listening and go/no-go tasks, especially for the alpha/beta ERD of central interest to this study.
(TIF)
