# Supplementary figures and images for: Auditory sensory processing induces cortical and thalamic event-related desynchronization in the mouse
Source: PLoS One. 2025 Oct 27;20(10):e0334293. doi: 10.1371/journal.pone.0334293 (PMC12558558; doi:10.1371/journal.pone.0334293)

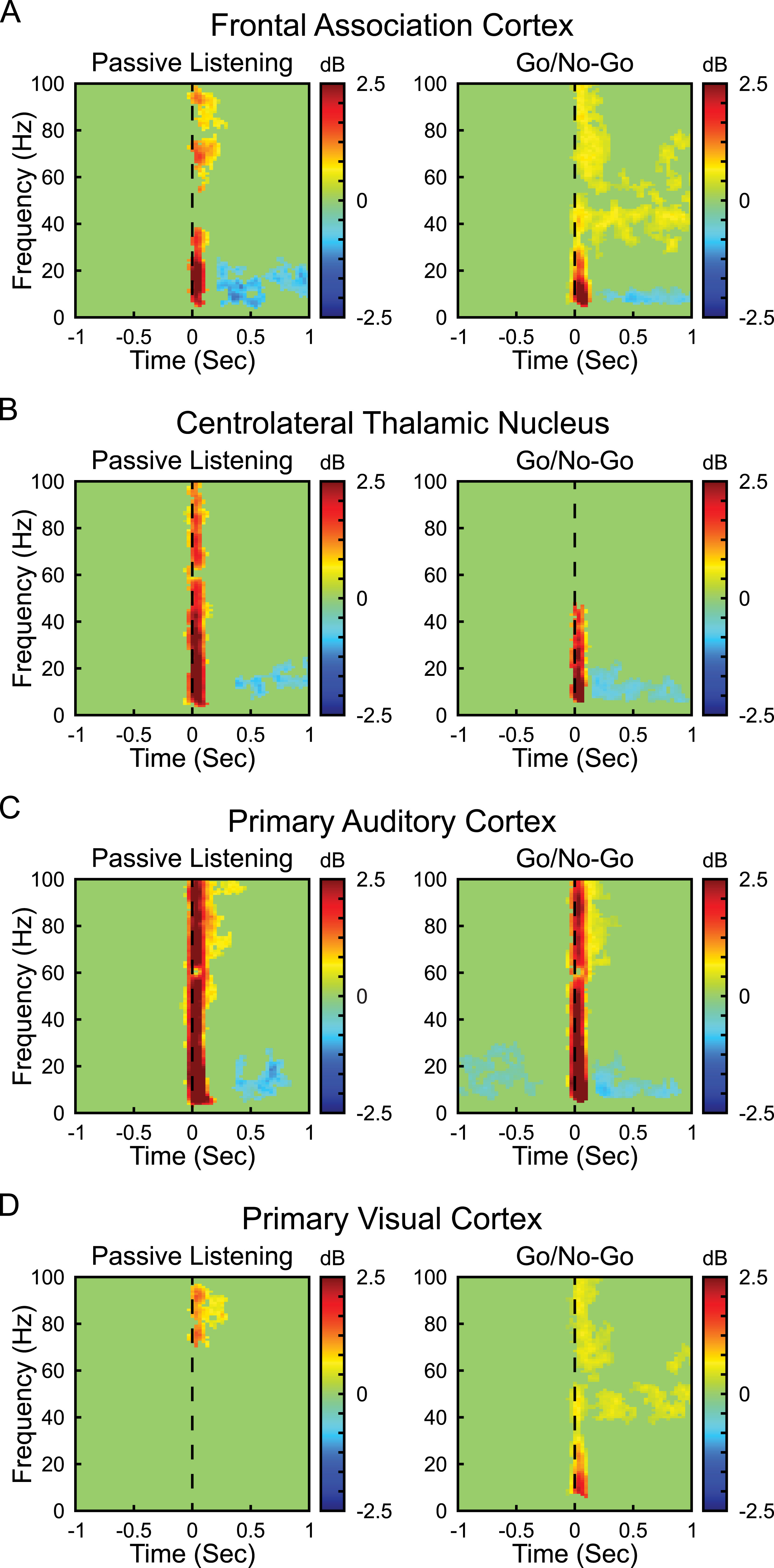

Supplement: S1 Fig — The differences between the mean peristimulus spectrograms of maximum-intensity auditory stimulus trials (65.7 dB) and no-stimulus trials (63.5 dB white noise only) are shown here for passive listening (left) and go/no-go (right tasks). Spectrograms time-locked to real or no-stimulus (blank) onset (time 0, vertical dashed line) were computed for all maximum-intensity and no-stimulus trials, then decibel-normalized to the prestimulus baseline (500ms before stimulus presentation). Peristimulus spectrograms were averaged across all trials of the same condition for each mouse, then the mean spectrogram across all mice was found for all trials of the same condition. The mean no-stimulus trial spectrograms were subtracted from the mean maximum-intensity trial spectrograms, and a cluster-based permutation testing was employed to identify significant differences between the conditions (p < 0.05, 5000 iterations). Warmer tones indicate increases in spectral power within maximum-intensity stimulus trials over no-stimulus trials, while cooler tones indicate decreases. (A) Frontal association cortex, (B) centrolateral thalamic nucleus, (C) primary auditory cortex and (D) primary visual cortex show few qualitative differences in between passive listening and go/no-go tasks, especially for the alpha/beta ERD of central interest to this study. (TIF) [file pone.0334293.s001.tif]
